# PPCD: Privacy-preserving clinical decision with cloud support

**DOI:** 10.1371/journal.pone.0217349

**Published:** 2019-05-29

**Authors:** Hui Ma, Xuyang Guo, Yuan Ping, Baocang Wang, Yuehua Yang, Zhili Zhang, Jingxian Zhou

**Affiliations:** 1 School of Information Engineering, Xuchang University, Xuchang, Henan, China; 2 No.1 Middle School of Zhengzhou, Zhengzhou, Henan, China; 3 Information Technology Research Base of Civil Aviation Administration of China, Civil Aviation University of China, Tianjin, China; 4 State Key Laboratory of Integrated Service Networks, Xidian University, Xi’an, China; Beijing University of Posts and Telecommunications, CHINA

## Abstract

With the prosperity of machine learning and cloud computing, meaningful information can be mined from mass electronic medical data which help physicians make proper disease diagnosis for patients. However, using medical data and disease information of patients frequently raise privacy concerns. In this paper, based on single-layer perceptron, we propose a scheme of privacy-preserving clinical decision with cloud support (PPCD), which securely conducts disease model training and prediction for the patient. Each party learns nothing about the other’s private information. In PPCD, a lightweight secure multiplication is presented and introduced to improve the model training. Security analysis and experimental results on real data confirm the high accuracy of disease prediction achieved by the proposed PPCD without the risk of privacy disclosure.

## Introduction

With sharp growth of electronic data, machine learning has impacted on human’s lifestyle by predicting human’s behavior and future trends on everything [1], [2], [3]. To overcome the limitations of storage and computing resource, how to outsource pricey tasks of machine learning to the Cloud has attracted much more attention. For instances, data of the client can be transmitted to the Cloud for either model training and predicting [4], [5], [6]. As a popular machine learning algorithm, single-layer perceptron (SLP) is simple yet efficient and has been widely used in disease prediction [7], [8], [9]. It is more appropriate for real-time disease predicting than some complex techniques such as naïve bayesian [10], decision trees [2] and support vector machines (SVMs) [11], [12] and so on. Clinical decision support system (CDSS), which uses various data mining techniques to help physicians make proper disease diagnosis and provide health services for patients, has received considerable attention [7], [13], [14],[15]. However, for privacy concerns, users don’t want to submit their medical data to an unauthorized institution [16], [17], [18]. At the same time, due to classifier being considered as own asset of the medical service provider, there is a risk of exposing the prediction model to third-party. Otherwise, third-party will use the model to make disease prediction for a patient who could damage the profile of medical service provider. Therefore, the confidentiality of both medical data and disease model are crucial for the CDSS. How to achieve secure disease prediction without compromising the accuracy of the result becomes a challenging issue.

**Fig 1:**
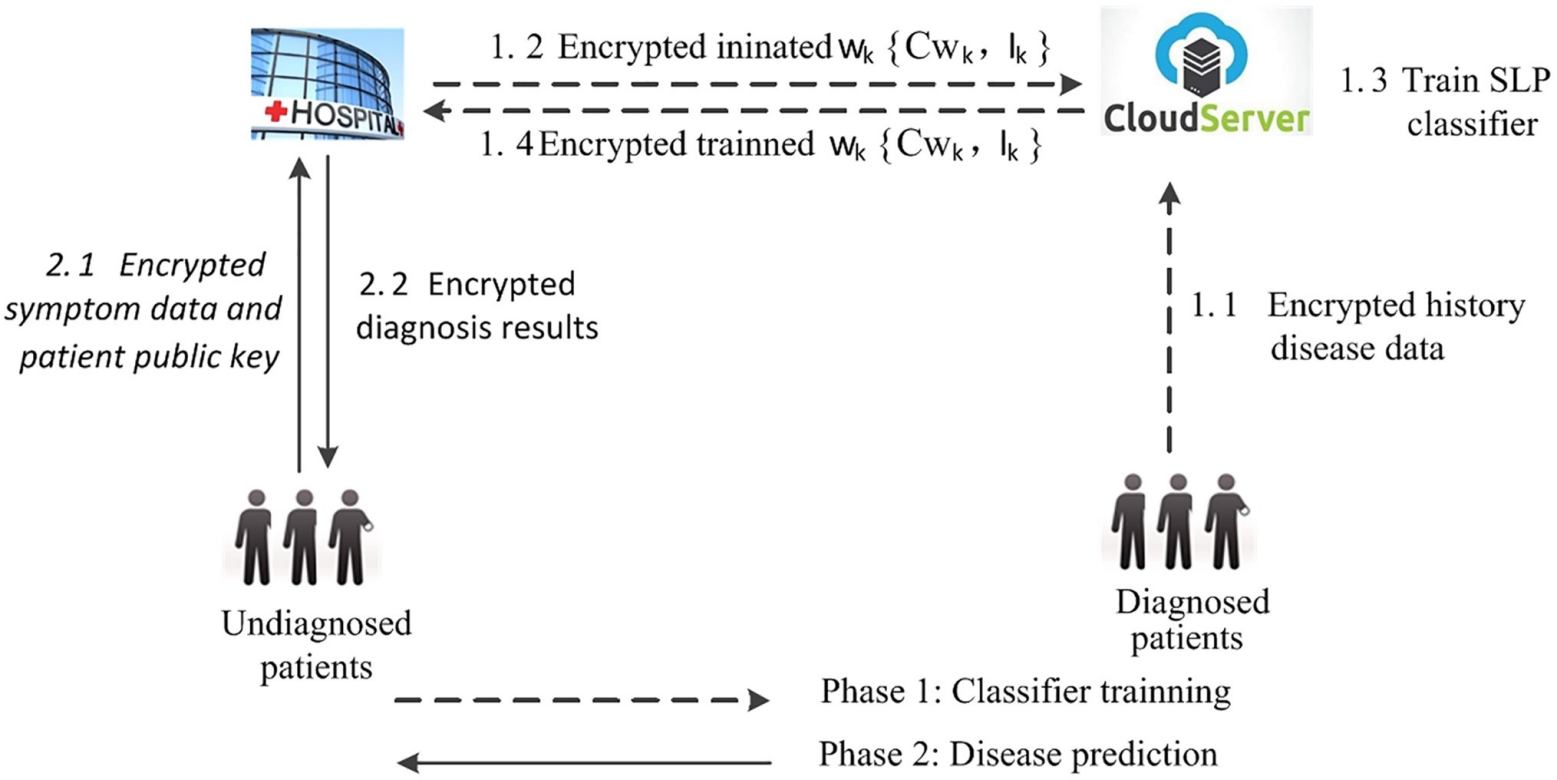
Architecture of the proposed PPCD.

To protect the privacy of patients’ medical data and the security of the prediction model, in this study, we propose a privacy-preserving clinical decision scheme based on SLP with cloud support (PPCD). As shown in Fig 1, two phases of SLP model training and disease predicting are included. In the model training, Diagnosed patients encrypt their symptoms data and outsource them with the corresponding diagnosed disease to the cloud. Meanwhile, the hospital generates random weights which are then encrypted and sent to the cloud. After receiving both of the encrypted medical data and the weights, the cloud trains the model accompanied by a few interactions with the hospital. The cloud selects an encrypted sample and executes the sign(.) function. If the returned value of sign(.) does not match its label, the cloud updates the weights until the convergence criterion is satisfied or all the disease cases are matched. When a patient wants to check his disease, he encrypts the data of the symptoms and submits it to the hospital which completes the analysis based on the disease model and sends back the encrypted diagnosis result and some medical advice.

Towards tackling the privacy concerns in Clinical decision support system, PPCD provides disease model training and disease risk prediction for the patient in a privacy-preserving way that makes the Cloud learns nothing about the patient’s medical information and the actual model. Specifically, the main contributions lie in:

1. The proposal of PPCD which provides a privacy-preserving clinical decision based on SLP with cloud support. It helps the doctor to predict disease since the medical data and the diagnosis result remains in encrypted forms. Furthermore, the built disease diagnosis model is also protected as an asset of the hospital.
2. For privacy-preserving in the phase of model training, a specific lightweight secure multiplication (LSM) is presented. By employing LSM, PPCD securely finishes the inner-product in encrypted-domain (ED) after one round.
3. We implement PPCD by Java to check its performance in ED. Experimental results from several medical data analysis confirm that PPCD achieves comparable accuracies with SLP in plain-domain (PD).

The remainder of this paper is organized as follows: The following section briefly introduces the preliminaries. Then, PPCD is proposed along with LSM. Also, correction & security analysis is detailed, followed by the section of performance evaluation. Related works and conclusions are respectively given by the last two sections.

## Preliminaries

In this section, a brief glimpse of the Paillier cryptosystem, SLP and secure multiplication (SM) are given. Table 1 summarizes the key notations.

**Table 1:** Summary of notations.

Notation	Definition
*PK*_*h*_	Hospital’s public key of the Paillier encryption scheme
*SK*_*h*_	Hospital’s private key of the Paillier encryption scheme
*PK*_*up*_	Undiagnosed Patient’s public key of the Paillier encryption
*SK*_*up*_	Undiagnosed Patient’s private key of the Paillier encryption
*$E_{PK_{h}}(\cdot )$*	The Paillier’s encryption function
$E_{SK_{h}}(\cdot )$	The Paillier’s decryption function
Sign(.)	Activation function of SLP
*x*_*i*_	Symptom vector of patient *i*
*O*_*i*_	Output value, *O*_*i*_ ∈ {−1, 1}
*D*_*k*_	The *k*-th disease, *k* ∈{1, *m*}
*$\vec{Cx_{i}}$*	Encrypted symptom vector of patient *i*
$\vec{CW_{k}}$	Weight ciphertext vector of *k*-th disease
*x*_*ij*_	The *j*-th symptom attribute of patient *i*
*Cx*_*i*,*j*_	Ciphertext of *x*_*ij*_
*Cw*_*j*_	Ciphertext of *w*_*j*_
|*x*_*ij*_|	The absolute value of *x*_*ij*_
*r*_*xij*_, *r*_*wj*_	The random numbers, *r*_*xij*_, *r*_*wj*_ ∈ *Z*_*N*_
*EXP*	Time cost of one exponentiation operation
*MUL*	Time cost of one multiplication operation
*DIV*	Time cost of one modular inverse operation
#	Not equal to

### Single-layer perceptron

Following [19], SLP is to learn the weight vector *w* which is then multiplied with the input features to determine if a sample belongs to one class or the other. We define an activation function *sign*(*z*) which takes the linear combination of the input values *x* and *w* as input. If *sign*(*z*) is greater than a defined threshold *θ*, we predict 1 and -1 otherwise. In order to simplify the notation, we define *w*_0_ = −*θ* and *x*_0_ = 1, so that

$$sign(z)=\left\{ \begin{array}{l} 1\;\text{if}\;z\geq \theta ,\\ -1\;\text{if}\;\text{otherwise}, \end{array}\right.$$ (1)

where

z=w0x0+w1x1+⋯+wnx=n∑i=0nwjxj=wTX.

For each training sample *x*_*i*_, we calculate the output value, and update *w* if the output is not the same with the target. The value for updating the weights at each increment is calculated by the learning rule,

$$w_{j+1}=w_{j}+\eta o_{i}x_{ij},$$ (2)

where *η* is the learning rate (0 < *η* ≤ 1).

It is important to note that the convergence of the perceptron is only guaranteed if the two classes are linearly separable. If a linear decision boundary can’t separate the two classes, a maximum number of passes should be set over the training dataset and/or a threshold for the number of tolerated misclassifications.

### Paillier cryptosystem

Paillier cryptosystem is an additively homomorphic cryptosystem [20]. It works as follows:

1. **Key generation**: Two large prime numbers *p* and *q* are randomly and independently chosen such that gcd(*pq*, (*p* − 1)(*q* − 1)) = 1, where |*p*| = |*q*|. Then, we compute *n* = *pq* and *λ* = *lcm*(*p* − 1, *q* − 1), and select a random integer *g* in $Z_{n^{2}}^{*}$. By setting *μ* = (*L*(*g*^*λ*^ mod *n*^2^))^−1^ mod *n* and $L(x)=\frac{x-1}{n}$, the public key (*n*, *g*) and the private key (*λ*, *μ*) are obtained.
2. **Encryption**: Let *m* be a message to be encrypted where 0 ≤ *m* < *n*. With a randomly selected *r* where 0 < *r* < *n*, the ciphertext is calculated by *c* = *E*(*m*) = *g*^*m*^ · *r*^*m*^ mod *n*^2^.
3. **Decryption**: Let *c* be the ciphertext to decrypt where $c\in Z_{n^{2}}^{*}$, the plaintext message is got by *m* = *D*(*c*) = *L*(*c*^*λ*^ mod *n*^2^) · *μ* mod *n*.

As a additively homomorphic, its identities: *D*((*E*(*m*_1_, *r*_1_) · *E*(*m*_2_, *r*_2_) mod *n*^2^) = (*m*_1_ + *m*_2_) mod *n* and homomorphic multiplication of plaintexts: *D*((*m*_1_, *r*_1_)^*k*^ mod *n*^2^) = *km*_1_ mod *n*.

#### Secure multiplication

Secure Multiplication(SM) [21] supports multiplication in ED. Suppose Alice has two encrypted data *E*_*pk*_(*X*) and *E*_*pk*_(*Y*), Bob has the private key *sk* corresponding to public key *pk*, the goal of SM is to compute *E*_*pk*_(*X* * *Y*) without leaking *X* and *Y* to Alice. SM protocol is described as follow:

1. Alice gets ciphertext *E*_*pk*_(*x*) and *E*_*pk*_(*y*), generates two random numbers *r*_*x*_, *r*_*y*_ ∈ *z*_*n*_, and then calculates *x*1 = *E*_*pk*_(*x*) · *E*_*pk*_(*r*_*x*_) and *y*1 = *E*_*pk*_(*y*) · *E*_*pk*_(*r*_*y*_). Send *x*1 and *y*1 to Bob.
2. After received *x*1 and *y*1, Bob decrypts *x*1 and *y*1 by using the private key *sk* to get *H*_*x*_ = *D*_*sk*_(*x*1) and *H*_*y*_ = *D*_*sk*_(*y*1), then computes *H*1 = *H*_*x*_ · *Hy* mod *N*, last Bob encrypts *H*1 with *pk H* = *E*_*pk*_(*H*1) and sends *H* to Alice.
3. Alice first computes $s1=E_{pk}{\left(x\right)}^{N}{}^{-r_{y}}$,$s2=E_{pk}{\left(y\right)}^{N-r_{x}}$ and *s*3 = *E*_*pk*_(*r*_*x*_ · *r*_*y*_)^*N*−1^, then multiplies them as *E*_*pk*_(*x* · *y*) = *H* · *s*1 ·*s*2 ·*s*3.

## The proposed PPCD model

### Model overview and requirements

#### Model overview

To make employing SLP for model training and disease prediction with privacy being protected, the proposed PPCD model contains four parties which are illustrated in Table 2. They collaboratively conduct SLP model training and disease predicting. The CS trains a disease prediction model based on the DP’s disease data. To check a disease, UP submits his symptoms data to the Hospital which predicts the corresponding disease based on the trained model. Fig 1 depicts the detailed procedure.

**Table 2:** Description of the attended four parties.

Parts	Descriptions
Diagnosed Patient(DP)	DP encrypts the symptoms data with the hospital’s public key *PK*_*h*_ and the diagnosed result, which are used for training disease model, and then outsources the data to the Cloud server
Undiagnosed Patients(UP)	UP provides the encrypted disease symptoms data for hospital to make decisions
Hospital	As a medical service provider, the hospital is a trusted party who is in charge of generating, distributing and management of public key and private key. Meanwhile, the hospital performs model training together with the cloud server and disease predicting for UP based on patient’s symptoms
Cloud Server (CS)	CS with almost unlimited storage trains the disease model according to the outsourced medical data. The trained model is securely stored in the hospital

#### Privacy requirements

In PPCD, DPs are trustworthy. They provide correct medical data to the Cloud server. Meanwhile, CS and UP are honest-but-curious [22]. CS strictly follows the privacy-preserving SLP learning protocol performed in the system. It wants to know HP’s sensitive medical data and UP’s medical information once the condition is met. UP is interested in the trained disease model. Hospital is honest. At the same time, an adversary from outside is curious about all transferred data in the system by eavesdropping. So privacy-preserving is critical for successfully diagnosing the patient’s disease, and security requirements of PPCD are listed as follows.

1. UP’s Privacy: In the disease diagnosis, sensitive symptom data of UP should not be leaked to other untrusted parties during the transmission. Furthermore, the diagnosed result is confidential for the patients such that it cannot be exposed to any other entities. It means that UP’s privacy should be preserved.
2. DP’s Privacy: Generally, DP gets some history medical information, e.g., the diagnosed disease and the confirmed symptoms data. This information is highly sensitive and cannot be got by the unauthorized entities. Otherwise, DP is unwilling to provide the history disease data for model training due to privacy concerns.
3. Hospital’s Privacy: In PPCD, hospital trains disease model using the historical medical data with the help of the Cloud. As an asset of the hospital, the disease model cannot be leaked to UP and other parties during disease diagnosis.

#### Design goal

Based on the above scenarios and the security requirements, the system will realize model training and disease diagnosis in a privacy-preserving and efficient way. The particular goals are shown as follows.

1. Privacy-preserving requirements: the flourish of Clinical decision support hinges upon information secure and privacy-preserving. If the model’s privacy requirements are not considered, the patient’s sensitive data and the disease model will be exposed to the unauthorized parties. Thus history patients are more unwilling to share their medical data to PPCD, the accuracy of the trained model is not ensured, and diagnosis service will be bad. Therefore, the system should realize the privacy of history patients and undiagnosed patients.
2. Confidentiality and accuracy of disease model should be achieved: the disease model is a valuable asset of the hospital, which may be reluctant to reveal the information of the disease model. Simultaneously, it is crucial applying privacy-preserving can’t compromise the accuracy of predicting model.

### The Proposed PPCD Model

#### Privacy-preserving training

This section shows how to construct PPCD, train the disease model and predict disease based on the model in a privacy-preserving way.

(1) System setting

**Key generation**: Paillier encryption algorithm is run by the hospital to generate keys for both UP and the hospital. Given the secure parameter *k*, choose two large prime numbers *p* and *q* randomly which satisfy |*q*| = |*p*| = *k*, hospital generates the pubic key (*n*, *g*) and the corresponding private key (*λ*, *μ*), where *n* = *pq* and *λ* = *lcm*(*p* − 1, *q* − 1).

**Data encryption:** Raw medical data xi,j∈xi→=(xi,1,xi,2,…,x)i,n are encrypted and submitted to the Cloud for storage and model training. The Cloud stores the disease patterns $<D_{k}{>}_{i=1}^{d}$, each of which represents a disease sample $<x_{i},O_{i}{>}_{i=1}^{m}$, where *x*_*i*_ is a *n*-dimension vector, each element represents confirmed symptom and *O*_*i*_ ∈ {−1, 1} is associated desired output, where 1 represents suffering from the disease and -1 represents not. Suppose medical data have been preprocessed, so the format of data is suitable for PPCD. In system, disease output is stored in cloud server in plaintext because leaking disease output does not damage patients’ privacy. The encrypted patients’ medical data are stored in cloud as Table 3.

**Table 3:** Medical data for the *k*-th disease.

Medical sample	Medical data	Desired output
*x*_1_	{*Cx*_1,1_, *Cx*_1,2_, ⋯, *Cx*_1,*n*_}	*O*1
*x*_2_	{*Cx*_2,1_, *Cx*_2,2_, ⋯, *Cx*_2,*n*_}	*O*_2_
⋯	⋯ ⋯	⋯ ⋯
*x*_n_	{*Cx*_*n*,1_, *Cx*_*n*,2_, ⋯, *Cx*_*n*,*n*_}	*O*_*i*_

Meanwhile, the disease predicting model is sensitive data which should be encrypted. At the beginning of model training, the hospital generates a random weight w = (w_1_, w_2_, ⋯, w_n_) and encrypts it, then sends ciphertext of the weight to the Cloud server.

(2) Lightweight secure multiplication protocol

SM can be used to calculate inner-product on the two encrypted vectors. Given $\vec{Cx_{i}}=(Cx_{i,1},Cx_{i,2},\ldots ,Cx_{i,n})$ and $\vec{CW}=(Cw_{1},Cw_{2},\ldots ,Cw_{n})$, $E(\sum_{i=1}^{n}x_{i}\cdot w_{i})$ is calculated by running SM for *n* times. To efficiently compute the inner-product of two encrypted vectors, based on SM, we propose an efficient lightweight secure multiplication (LSM) protocol which can achieve inner-product on ciphertext in one time. By considering two parties *C*1 and *C*2, LSM is detailed in Algorithm 1.

**Algorithm 1**: $LSM(\vec{Cx_{i}},\vec{W})\to E(\sum_{i=1}^{n}x_{i}\cdot w_{i})$

**Require**: *C*1 has $\vec{Cx_{l}}$ and $\vec{CW}$; *C*2 has *sk*

Step1: *C*1:

(1) Chooses 2*n* random numbers *r*_*xij*_, *r*_*wj*_, ∈ *Z*_*N*_
(2) *Cr*_*xij*_ ← E(*r*_*xij*_)
(3) *Cr*_*wj*_ ← *E*(*r*_*wj*_)

For each *Cx*_*ij*_ and *Cw*_*j*_

(4) X_ij_ = Cx_ij_ · Cr_xij_
(5) *W*_*j*_ = *Cw*_*j*_ · *Cr*_*wj*_; Send *X*_*ij*_, *W*_*j*_ to the *C*2

Step2: *C*2

(1) Receive *X*_*ij*_ *W*_*j*_ from *C*1
(2) $X^{\prime }{}_{ij}\leftarrow D_{sk}(X_{ij})$
(3) $W^{\prime }{}_{j}\leftarrow D_{sk}(w_{j})$
(4) $h=\sum_{i=1}^{n}X^{\prime }{}_{ij}\cdot w^{\prime }{}_{j}$
*(5) H* = *E*_*pk*_(*h*); sends *H* to *C*1

Step3: *C*1

(1) Receiving the *H*
(2) $T1=\prod_{i=1}^{n}E{\left(r_{xij}\cdot r_{wj}\right)}^{N-1}$
(3) $T2=\prod_{i=1}^{n}E{\left(x_{ij}\right)}^{N-r_{wj}}$
(4) $T3=\prod_{i=1}^{n}E{\left(w_{ij}\right)}^{N-r_{xij}^{}}$
(5) $R=H\cdot T_{1}\cdot T_{2}\cdot T_{3}=E(\sum_{i=1}^{n}x_{i}\cdot w_{i})$

(3) Model training

In system setting phase, DP encrypts its medical information <*x*_*i*_, *O*_*i*_> and outsources <*Cx*_*i*_, *O*_*i*_> to the Cloud. The Cloud collects some medical data $<Cx_{i},O_{i}{>}_{i=1}^{m}\in D_{k}$ where *k* represents the *k*-th disease. To train the predicting model *w*_*k*_ of the *k*-th disease, the Cloud selects disease samples with *I*_*k*_ to train the model.

Privacy-preserving disease model training is described by Algorithm 2.

**Algorithm 2**: **Privacy-Preserving Model Training Based on SLP**

1: **Input**: *n* input samples, $<Cx_{i},O_{i}{>}_{i=1}^{n}\in <D_{k}{>}_{k=1}^{m}$, 1 ≤ *k* ≤ *m*, *iteration*_*max*_, learning rate *η*, sign function *sign*(·)

2: **Output**: prediction model *w*_*k*_, 1 ≤ *k* ≤ *m*

3: **DP:** for 1 ≤ *k* ≤ *m* do

4: for 1 ≤ *i* ≤ *n* do

5:  DP encrypts symptom data as <*Cx*_*i*_, *O*_*i*_, *I*_*k*_> and submits to the cloud

6:   Endfor

7:   Endfor

8: for 1 ≤ *k* ≤ *m* do

9: **Hospital:** chooses initialization $\vec{w_{k}}$ randomly.

10:   for *iteration* = 1, 2, …, *iteration*_max_

11:    for 1 ≤ *i* ≤ *n* do

12: **Hospital:** encrypts $\vec{w_{k}}$ and upload to the cloud

13: **Cloud:** chooses a medical sample <*Cx*_*i*_, *O*_*i*_> and executes *LSM* to get

14:    $R=E(\sum_{j=1}^{d}x_{ij}\cdot w_{j})$ and send to the hospital

15: **Hospital:** decrypt *R* and calculation sign function *Si* = *sign*(*DEC*(*R*)) and send to the cloud.

16: **Cloud:** If *S* # *O*_*i*_ and *O*_*i*_ = 1, exp = *η*

17:   If *S* # *O*_*i*_ and *O*_*i*_ = −1, exp = *n* − *η*

18:    for *j* = 1,…*d*

19:     $u_{j}=Cx_{i,j}^{\text{exp}}$

20:     *Cw*_*j*_ = *Cw*_*j*_ ⋅ *u*_*j*_

21:    endfor

22:   endfor

23:  endfor

24: return *w*_*k*_, 1 ≤ *k* ≤ *m*

Lines **3–7:** DP encrypts symptom data and submits <*Cx*_*i*_, *O*_*i*_, *I*_*k*_> to the cloud.

Lines **8–12:** The hospital randomly generates the weight $\vec{w_{k}}$ in which not all elements is equal to 0 and encrypts it with own public key *pk*, then, send weight ciphertext $\left\{\vec{Cw_{k}},I_{k}\right\}$ {to the Cloud.

Lines **13–14:** In the Cloud, choose a disease sample {*C*_*xi*_, *I*_*k*_} and 2*n* random numbers *r*_*xij*_, *r*_*wj*_ ∈ *Z*_*N*_, then executes *LSM* to compute $R=E(\sum_{i=1}^{n}x_{ij}\cdot w_{i})$, where the cloud server is C1, hospital is C2. Lastly send *R* to the hospital.

Lines **15:** After receiving *R*, teh hospital decrypts *R* with private key *sk*, and execute the *sign*(·) function as $S=sign(\sum_{i=1}^{n}x_{i}\cdot w_{i})$, then send *S* to cloud.

Lines **16–20:** The Cloud compare *S* with *O*_*i*_. if S # *O*_*i*_ and *O*_*i*_ = 1, let exp = *η*; if S # *O*_*i*_ and *O*_*i*_ = −1, let exp = *n* − *η*. Next the Cloud updates *C*_*xi*_ as $Cx_{i,j}{}^{\text{exp}}$, and then, update *Cw*_*j*_ as $Cw_{j}\cdot Cx_{i,j}{}^{\text{exp}}$.

**Line 24:** If the entire disease samples are matched or training count is greater than convergence criterion, hospital will terminate the training model and <*w*_*k*_ *I*_*k*_> is seen as prediction model for *D*_*k*_, else return and repeat lines **13–14**.

After getting the *k*-th disease model, the Cloud selects $<Cx_{i},O_{i}>\sum_{i=1}^{m}\in D_{k+1}$ and repeats lines **8–24**. After all medical sample are trained, hospital cloud get prediction models $<Cw_{k},I_{k}{>}_{k=1}^{m}$ for all disease.

#### Disease prediction

In the phase, assuming prediction models have been trained and stored in the hospital. The hospital can predict whether a patient suffers from *K*-th disease using a *K*-th disease model. When an undiagnosed patient submits his encrypted symptoms information to the hospital, the prediction will be executed as follow.

**Step 1:** When the ciphertext of symptoms information is arrived, the hospital decrypts the ciphertext and gets the plaintext symptoms data $\vec{x_{i}}$.
**Step 2:** Let *s* = 0, for each *x*_*j*_ and *w*_*j*_, the hospital calculates *s*_*j*_ = *x*_*j*_ · *w*_*j*_, then gets $s=\sum_{j=1}^{n}s_{j}$.
**Step 3:** Compute *S* = *sign*(*s*), If S > = 0, then the patient suffers from the disease, but not otherwise.
**Step 4:** hospital encrypts the prediction result with UP’s public key and return to the patient.

## Correction & security analysis

In this section, we analyze the correction and security of the proposed PPCD scheme. Notably, we focus on how PPCD achieve the privacy preserving of medical information of patient and disease model.

### (1) Correctness analysis of LSM

The correctness of LSM can be illustrated as follows:

In Step1:

$$X_{ij}=Cx_{ij}\cdot E(r_{xij})=E(x_{ij})\cdot E(r_{xij})=E(x_{ij}+r_{xij})$$ (3)

$$W_{j}=Cw_{j}\cdot E(r_{wj})=E(w_{j})\cdot E(r_{wj})=E(w_{j}+r_{wj})$$ (4)

In Step2:

$$X_{ij}{}^{\prime }=D_{sk}(X_{ij}),\;W_{j}{}^{\prime }=D_{sk}(w_{j})$$ (5)

h=∑i=1nXij′⋅wj′=∑i=1n(xi+rxi)(w+irwi) (6)

$$H=E_{pk}(h)=E(\sum_{i=1}^{n}\left(x_{i}^{}+r_{xi})(w_{i}^{}+r_{wi}\right))$$ (7)

In the Step3:

$$T_{1}=\prod_{i=1}^{n}E{\left(r_{xij}\cdot r_{wj}\right)}^{N-1}=\prod_{i=1}^{n}E(-r_{xij}\cdot r_{wj})=E(\sum_{i=1}^{n}-r_{xij}\cdot r_{wj})$$ (8)

$$T_{2}=\prod_{i=1}^{n}E{\left(x_{ij}\right)}^{N}{}^{-r_{wj}}=\prod_{i=1}^{n}E(-r_{wj}\cdot x_{ij})=E(\sum_{i=1}^{n}-r_{wj}^{}\cdot x_{ij})$$ (9)

$$T_{3}=\prod_{i=1}^{n}E(w_{j})N-r_{xij}=\prod_{i=1}^{n}E(-r_{xij}\cdot w_{j})=E(\sum_{i=1}^{n}-r_{xij}\cdot w_{i})$$ (10)

$$\begin{array}{l} R=H\cdot T_{1}\cdot T_{2}\cdot T_{3}\\ \;=E(\sum_{i=1}^{n}\left(x_{i}\cdot w_{i}+x_{i}\cdot r_{wi}+r_{xi}\cdot w_{i}+r_{xi}\cdot r_{wi}-r_{wi}\cdot x_{i}-r_{xi}\cdot w_{i}-r_{xi}\cdot r_{wi}\right))\\ \;=E(\sum_{i=1}^{n}x_{i}\cdot w_{i}) \end{array}$$ (11)

From the above derivation, *LSM* can calculate the $E(\sum_{i=1}^{n}x_{i}\cdot w_{i})$ in a round.

### (2) Correctness analysis of training model

The correctness of PPCD can be illustrated as follows: in step3, the hospital decrypts *R* with private key *sk*, and compute

$$s_{i}=sign(Dec(R))=sign(Dec(E(\sum_{j=1}^{d}x_{i}{}_{j}\cdot w_{j})))=sign(\sum_{i=1}^{n}x_{ij}\cdot w_{i})=sign(w_{k}\cdot x_{i}{}^{T})$$ (12)

So *s*_*i*_ is consistent with that in Eq (1).

In Step 4. The Cloud update *Cw*_*k*_ as *Cw*_*j*_ = *Cw*_*j*_ · *u*_*j*_,

where $u_{j}=Cx_{i,j}{}^{\text{exp}}$

If *S* # *O*_*i*_ and *O*_*i*_ = 1, exp = *η*

$$u_{j}=Cx_{i,j}{}^{\text{exp}}=Cx_{i,j}{}^{\eta }=E(x_{i,j}\cdot \eta )$$ (13)

Then

$$Cw_{j}=Cw_{j}\cdot u_{j}=E(w_{j})\cdot E(x_{i,j}^{}\cdot \eta )=E(w_{j}+x_{i,j}\cdot \eta )=E(w_{j}+\eta \cdot O_{i}x_{i,j})$$ (14)

If *S* # *O*_*i*_ and *O*_*i*_ = −1, exp = *n* − *η*

$$u_{j}=Cx_{i,j}{}^{\text{exp}}=Cx_{i,j}{}^{n-\eta }=E(-x_{i,j}\cdot \eta )$$ (15)

Then

$$Cw_{j}=Cw_{j}\cdot u_{j}=E(w_{j})\cdot E(-x_{i,j}\cdot \eta )=E(w_{j}-x_{i,j}\cdot \eta )=E(w_{j}+\eta \cdot O_{i}x_{i,j})$$ (16)

Thus *Cw*_*j*_ is also consistent with that in Eq (2).

From the above calculation, PPCD train correct disease model in the cloud. Namely the accuracy of prediction model is satisfied.

### (3) Security of patient’s medical data

To predict disease for patients, DP and UP encrypt medical information *x*_*i*_ = {*x*_*i*1_, *x*_*i*2_,…,*x*_*ij*_} with the hospital’s public key *PK*_*h*_ and upload the ciphertext *Cx*_*i*_ = {*Cx*_*i*1_, *Cx*_*i*2_,…,*Cx*_*ij*_} to the Cloud. In the process of transmission, all the medical information is encrypted to prevent outside attacker from eavesdropping. An adversary cannot decrypt the ciphertext without the hospital’s private key *SK*_*h*_. The symptom data is encrypted by the Paillier which is semantic secure against the choose plaintext attack. So the medical information stored in the Cloud is secure since the Cloud cannot identify the corresponding contents and get the plaintext of symptom data.

### (4) Security of training disease model

During training the prediction model, all the computations are done over ciphertexts. $E(\sum_{i=1}^{n}x_{ij}\cdot w_{i})$ is calculated by using *LSM* in which each party learns nothing from the protocol. The initial model is generated by the hospital randomly and updated in the process of training over ciphertext, and the hospital’s *SK*_*h*_ is well protected. $Cx_{i,j}{}^{\text{exp}}$ and *Cw*_*j*_ = *Cw*_*j*_ · *u*_*j*_ = *E*(*w*_*j*_ + *ηO*_*i*_*x*_*i*,*j*_) can be computed easily over ciphertext because of the additive homomorphism property of Paillier. Suppose the disease model is leaked to UP or the Cloud, they are not able to recover *w*_*k*_, without the private key *SK*_*h*_.

### (5) Security of predicting result

When a patient wants to identity his disease, he submits the ciphertext of symptoms data to the hospital. After finishing disease prediction, diagnosis result is encrypted by UP’s public key *PK*_*up*_ and returned to *UP*. When an attack captures predicting result, he can’t recover the corresponding contents without DP’s private key *SK*_*up*_.

## Performance evaluation

### Complexity analysis

#### Computational complexity

To analyze the complexity of the proposed PPCD, Table 4 illustrates the computational cost for each step. For simplicity, we use *EXP* to denote the time complexity of one exponentiation operation on ciphertext in the Paillier cryptosystem. Similarly, the time complexities of one multiplication operation on ciphertext and one modular inverse operation in the decryption algorithm are represented by *MUL* and *DIV*, respectively. In Step 1 of the disease learning phase, *n* exponents and multiplications are required by the hospital which encrypts the initial weight. In Step 2, the Cloud uses (2*n*+3) exponents and (4*n*+7) multiplications, and the hospital executes 2*n* exponents and 4n multiplications to obtain *R*. In Step 3, one exponent and one modular inverse are consumed before getting *S*. In Step 4, to update the weight, the Cloud does *n* exponents and *n* multiplication. At last, (*n*-1) multiplications, one exponent and one modular inverse are executed to predict disease risk. Then the encrypted diagnosis result is sent to UP.

**Table 4:** Summary of computational cost for *x*_*i*_ in PPCD.

Phase	Step	Entity	Computational cost
Disease learning	Step 1	Hospital	*n*(*EXP*+*MUL*)
	Step 2	Cloud	(2*n*+3)*EXP*+(4*n*+7)*MUL*
		Hospital	2*n*(*EXP*+2*MUL*)
	Step 3	Hospital	*EXP*+*DIV*
	Step 4	Cloud	*n*(*EXP*+*MUL*)
Disease prediction	Step 1	Hospital	(*n*-1)*MUL*+*EXP*+*DIV*

#### Communication complexity

Assuming there are *N* samples with n dimensions, and the length of the ciphertext is *p*. In the proposed PPCD system, the encrypted symptom data are outsourced to the Cloud to train the classifier which costs O(*N*(*np*+*L*)). In model training, the hospital transmits the encrypted initial weight which requires O(*np*+*L*_*IK*_). To compute *R*, the cost of transferring data is O(3*np*+2*p*+*L*_*IK*_). In disease prediction, the hospital sends the encrypted predicting result to UP that costs O(*np*+*L*_*IK*_). The communication complexities of the proposed PPCD are detailed in Table 5.

**Table 5:** Summary of communication overhead in PPCD.

Phase	Step	Communication overhead
Outsourcing DP’s data		*N*(*np*+*L*)
Disease learning	Step 1	*np*+*L*_*IK*_
	Step 2	2*np*+2*p*
	Step 4	*np*+ *L*_*IK*_
Disease prediction		*np*+ *L*_*IK*_

### Experimental results

To fairly evaluate the performance, the proposed PPCD is implemented by Java on Windows 7-X64. The Cloud is a computer with Intel Quad core 3.4GHz and 16GB available RAM, the hospital runs a machine with Intel Quad core 3.4GHz and 8GB available RAM, and the patient uses a laptop with Intel Dual core 2.0GHz and 8GB available RAM.

#### Data sets

In the experiment, we use the Wisconsin breast cancer dataset (WBCD), the heart disease dataset (HDD) and the acute inflammations dataset (AID) from the UCI machine learning repository [23] to test the performance of SLP based on our PPCD scheme. Table 6 shows the statistical information of the employed three datasets.

**Table 6:** Description of the benchmark data sets.

Data sets	size	dims	#classes	attributes
WBCD	683	9	2	clump thickness; uniformity of cell size; uniformity of cell shape; marginal adhesion; single epithelial cell size; bare nuclei; bland chromatin; normal nucleoli; mitoses
HDD	297	13	2	age; sex; cp; trestbpl; chol; fbs; restecg; thalach; exang; oldpeak; slope; ca; thal
AID	120	6	2	temperature; occurrence of nausea; lumbar pain; urine pushing; micturition pains; burning of urethra, itch, swelling of urethra outlet

WBCD contains 683 instances, and each instance includes 9 attributes ranging from 1 to 10. In WBCD, each instance can be grouped into one of two possible classes: benign or malignant. HDD has 297 instances, and each instance consists of 13 attributes with two classes. Except for sex, trestbpl, chol and thalach, the other 9 attributes range from 1 to 10. AID contains 120 instances, and each instance includes 6 attributes with two decisions, i.e., inflammation of urinary bladder (IUB) and nephritis of renal pelvis origin (NRPO). Except for the temperature, the other attribute is either 1 (YES) or 0 (No).

In reality, the raw medical data $x_{i,j}\in \vec{x_{i}}=(x_{i,1},x_{i,2},\ldots ,x_{i,n})$ may be decimal. However, the Paillier can only encrypt integers. To resolve the above problem, approximation and expansion (A&E) method is adopted. Following the suggestion of [12], we adopt expanding each piece of medical data by multiplying 10^4^, and rounding off all the values after the decimal point. For instance, *x*_*ij*_ is an integer lying in (*Z*_*n*_ ∼ −*Z*_*n*_), the item of weight *w* = (*w*_1_, *w*_2_, …, *w*_*n*_) is in (*Z*_*n*_ ∼ −*Z*_*n*_), then *x*_*i*,*j*_ are encrypted using the Pallier as follows.

$$\begin{array}{l} Cx_{i,j}=\left\{ \begin{array}{l} E(x_{i,j})\;x_{i,j}\geq 0,\\ E(n-\left|x_{i,j}\right|)\;x_{i,j}<0, \end{array}\right. \end{array}$$ (17)

$$Cw_{j}=\left\{ \begin{array}{l} E(w_{j})\;w_{j}\geq 0,\\ E(n-\left|w_{j}\right|)\;w_{j}<0, \end{array}\right.$$ (18)

where *Cx*_*i*,*j*_, *Cw*_*j*_ are the ciphertexts of *x*_*i*,*j*_ and *Cw*_*j*_, respectively.

#### Results and analysis

We conduct PPCD with a predefined iteration threshold 100, and then use the classifier and three real data sets to evaluate the classifier’s performance in terms of accuracy. For each data set, the ratio of training data samples to the testing data samples is 7:3. Experimental results are detailed in Tables 7–10. Apparently, for breast cancer, the overall accuracy achieved by SLP is 96.2% while PPCD reaches 95.6%. For heart disease, SLP obtains an overall accuracy of 94.6%, and PPCD has 93.9%. On AID, SLP gets an accuracy of 93.3% for IUB while PPCD achieves a comparable result 92.5%. For NRPO in AID, accuracy for SLP is 93.3% while PPCD gets 91.7%. Actually, PPCD reaches comparable disease analysis results with that of by SLP.

**Table 7:** Accuracy comparisons of SLP in PD and PPCD in ED on WBCD.

Output/Target		Class 1	Class 2	Overall
SLP(PD)	Class 1	426(62.3%)	18(2.6%)	96.0%
	Class 2	8(1.2%)	231(33.8%)	96.7%
	Overall	98.2%	92.8%	96.2%
PPCD(ED)	Class 1	423(61.9%)	21(3.1%)	95.3%
	Class 2	9(1.3%)	230(33.7%)	96.2%
	Overall	97.9%	91.6%	95.6%

**Table 8:** Accuracy comparisons of SLP in PD and PPCD in ED on HDD.

Output/Target		Class 1	Class 2	Overall
SLP(PD)	Class 1	155(52.2%)	5(1.7%)	96.9%
	Class 2	11(3.7%)	126(42.4%)	92.0%
	Overall	93.4%	96.2%	94.6%
PPCD(ED)	Class 1	155(52.2%)	5(1.7%)	96.9%
	Class 2	13(4.4%)	124(41.8%)	90.5%
	Overall	92.3%	96.1%	93.9%

**Table 9:** Accuracy comparisons of SLP in PD and PPCD in ED for IUB of AID.

Output/Target		Class 1	Class 2	Overall
SLP(PD)	Class 1	57(47.5%)	2(1.7%)	96.7%
	Class 2	6(5%)	55(45.8%)	90.2%
	Overall	90.5%	96.5%	93.3%
PPCD(ED)	Class 1	55(45.8%)	4(3.3%)	93.2%
	Class 2	5(4.2%)	56(46.7%)	91.8%
	Overall	91.7%	93.3%	92.5%

**Table 10:** Accuracy comparisons of SLP in PD and PPCD in ED for NRPO of AID.

Output/Target		Class 1	Class 2	Overall
SLP(PD)	Class 1	48(52.2%)	2(1.7%)	96.0%
	Class 2	6(3%)	64(42.4%)	91.4%
	Overall	88.9%	97%	93.3%
PPCD(ED)	Class 1	46(52.2%)	4(1.7%)	92%
	Class 2	6(4.4%)	64(41.8%)	91.4%
	Overall	88.5%	94.1%	91.7%

In terms of efficiency, Table 11 gives the runtime comparisons of PPCD on the three data sets. For Breast cancer, it takes 6.125s for history patients to encrypt all the symptoms. In the training phase, it takes 2993.1s for the Cloud to train the classifier. In the predicting phase, it takes 0.098s for the hospital to computer undiagnosed patient’s disease risk (including 0.013s for UP to encrypt all the symptoms). For Heart disease and AID, the time cost of data encryption, model training, and disease predicting are decreased as the reduction of the number of sample cases. For the sake of simplicity, multicore programming has not adopted the evaluation.

**Table 11:** Runtime comparisons of PPCD in ED and SLP in PD.

Dataset	Phase	PPCD(s)	SLP(s)
Breast cancer	Data encryption	6.125	---
	Model training	2993.100	0.012
	Disease predicting	0.098	0.005
Heart disease	Data encryption	3.259	----
	Model training	1860.505	0.010
	Disease predicting	0.145	0.002
AID(UIB)	Data encryption	1.564	---
	Model training	743.875	0.010
	Disease predicting	0.143	0.001
AID(NRPO)	Data encryption	1.467	---
	Model training	683.387	0.080
	Disease predicting	0.148	0.001

Note: "---" means not available.

## Related work

Without sufficient storage, computation or knowledge of the clinical decision, the clients frequently prefer outsourcing their data to the Cloud for model training and disease predicting. Ledley and lusted [24] firstly proposed a clinical decision support system which can help physicians to solve diagnostic problems. Later, a large number of disease prediction system based on various data mining techniques have been presented. For example, a fast prediction disease system based on SVM was proposed by [25] to predict the risk of progression of adolescent idiopathic scoliosis. Wang et al. [26] gave a risk assessment for individuals with a family history of pancreatic cancer using Bayesian classification. By introducing SVM, Huang et al. [27] designed a prediction model for breast cancer diagnosis while Barakat et al. [28] focused on the diagnosis of diabetes mellitus. For heart disease analysis, Anooj et al. [29] tried to use specific fuzzy rules. Though various prediction models have been developed, privacy protection of patients medical information fails to take into account which will impede the more progress of CDSS.

To address this challenge, some secure disease prediction [1], [7], [8], [9], [11], [12], [14] which diagnose patients’ disease without leaking medical data and prediction model have been widely studied. Wang et al. [14] proposed a Healer framework based on somewhat homomorphic encryption. It uses a small samples size to facilitate secure rare variants analysis and obtains the final results by decrypting ciphertexts in the trusted party. A privacy-preserving CDSS on Naïve Bayesian Classification was proposed by Liu et al. [5] which can help a clinician to diagnose the risk of patients’ disease in a privacy-preserving way. Wang et al. [9] proposed a secure SLP learning model for e-Healthcare, but it can only protect the privacy of patients’ medical information, the disease model isn’t protected. In [11], Zhu et al. proposed an efficient and privacy-preserving medical pre-diagnosis framework using SVM which can protect the sensitive personal health information without privacy disclosure with lightweight multi-party random masking and polynomial.

Recently, Tsung et al. [30] proposed a decentralized privacy-preserving healthcare predictive modeling framework on private Blockchain networks, in which privacy-preserving online machine learning is integrated with a private Blockchain network, apply transaction metadata to disseminate partial models, and design a new proof-of-information algorithm to determine the order of the online learning process, Each participating site contributes to model parameter estimation without revealing any patient health information. Zhang et al. [1] proposed a secure disease prediction scheme based on matrices and SLP which builds on new medical data encryption, disease learning, and disease prediction algorithms that utilizes random matrices. Liu et al. [7] proposed a Hybrid privacy-preserving clinical decision support system in fog–cloud computing, in which a fog server uses SLP to securely monitor patients’ health condition in real-time, The newly detected abnormal symptoms can be further sent to the cloud server for high-accuracy prediction in a privacy-preserving way. Compared with some sophisticated machine learning algorithms such as Naïve Bayesian, SVM, and deep learning classification, SLP is efficient and straightforward.

## Conclusions

In this paper, we proposed a privacy-preserving disease predicting system based SLP which can help physicians make a proper diagnosis of disease and provide health services for patients anytime anywhere in a privacy-preserving way. In PPCD, DP’s historical medical data are used to train SLP in ED, and the hospital uses the trained model to predict diseases for a UP. Towards easing the privacy concerns from DP, we suggest an additively homomorphic encryption also for simplicity and generality. Inevitable multiplications of SLP motivate us introducing LSM into PPCD. Then users’ medical information and the trained model are secret to the cloud. Compared with SLP, comparable results reached by PPCD suggest that sacrificing data precision to improve efficiency is feasible in practical use.

Although PPCD benefits privacy-preserving diagnosis, the balance between security and efficiency should be considered firstly. Therefore, how to optimize the model training using mini-batch for efficiency improvement and finding an effective way of introducing some other advanced machine learning methods to build the privacy-preserving disease prediction system are worthy of investigation.
